# Predictors of distant, local and lymph node recurrence for surgically treated non-small cell lung cancer (NSCLC) patients: Retrospective analysis of pathological T1–4, N0, R0, M0 tumor stages

**DOI:** 10.1055/a-2766-4308

**Published:** 2026-01-22

**Authors:** Julia Zimmermann, Julia Walter, Nicole Samm, Gökce Yavuz, Christian Ketscher, Niels Reinmuth, Martina Merk, Rudolf Hatz, Amanda Tufman, Christian P. Schneider

**Affiliations:** 1 Thoracic Surgery 9183 Ludwig-Maximilians-Universität MünchenMunichGermany; 2 Thoracic Surgery 9160 Asklepios Fachkliniken München-GautingGautingBYGermany; 3 Internal Medicine V 9183 Ludwig-Maximilians-Universität MünchenMunichGermany; 4 Thoracic Oncology 9160 Asklepios Fachkliniken München-GautingGautingGermany; 5 Institute for Lung Health and Immunity and Comprehensive Pneumology Center with the CPC-M bioArchive 167561 Helmholtz Zentrum München Institut für Molekulare ImmunologieMunichBYGermany

**Keywords:** non-small cell lung cancer, postoperative recurrence, risk factors, tumor size, diffusing capacity for carbon monoxide, nicht-kleinzelliges Lungenkarzinom, postoperatives Rezidiv, Risikofaktoren, Tumorgröße, Diffusionskapazität für Kohlenmonoxid

## Abstract

**Background:**

Lung cancer has a high recurrence rate after successful surgical treatment. This study deals with possible risk factors and recommendations to improve the treatment of non-small cell lung cancer (NSCLC).

**Methods:**

In this retrospective analysis, we used data of all NSCLC patients who underwent lobectomy at the Lung Cancer Center Munich between 2011 and 2020. Only patients with postoperative T1–4, N0, R0, M0 were included. We compared numerical outcomes between patients with distant, lymph node, local recurrence and no recurrence using analysis of variance (ANOVA), and categorical outcomes using Chi-squared test or Fisher’s exact test when cell numbers were <6. We used logistic regression models to identify factors significantly associated with the occurrence of a distant, lymph node and local recurrence.

**Results:**

Tumor size in cm was significantly higher in patients with local recurrence (mean 5.5) followed by distant recurrence (mean 4.0), lymph node recurrence (mean 3.1) and patients with no recurrence (mean 3.0), p<0.0001. Diffusing capacity for carbon monoxide (DLCO) in % was significantly higher in patients with no recurrence (72.9) and decreased with distant recurrence (67.0), local recurrence (66.7) and lymph node recurrence (65.8), p=0.01. There were no significant differences in postoperative complications, surgical approach or number of lymph node assessed. A tumor size ≥4.65 cm was identified as an independent marker for local recurrence.

**Conclusion:**

For patients with NSCLC ≥4.65 cm, which corresponds to UICC stage IIA and higher, multimodal therapy should be discussed. The surgical approach has no influence on recurrence.

## Zusammenfassung

**Hintergrund:**

Lungenkrebs weist nach erfolgreicher chirurgischer Behandlung eine hohe Rezidivrate auf. Diese Studie befasst sich mit möglichen Risikofaktoren und Empfehlungen zur Verbesserung der Behandlung des nicht-kleinzelligen Lungenkarzinoms (NSCLC).

**Methoden:**

In dieser retrospektiven Analyse wurden die Daten aller NSCLC-Patienten verwendet, die sich zwischen 2011 und 2020 einer Lobektomie am Lungenkrebszentrum München unterzogen. Es wurden nur Patienten mit postoperativem T1–4, N0, R0, M0 eingeschlossen. Wir verglichen numerische Ergebnisse zwischen Patienten mit Fernmetastasen, Lymphknotenrezidiven, Lokalrezidiven und rezidivfreien Patienten mithilfe der Varianzanalyse (ANOVA) und kategorische Ergebnisse mithilfe des Chi2-Tests oder des exakten Fischertests, wenn die Zellzahlen <6 waren. Mit logistischen Regressionsmodellen wurden Faktoren ermittelt, die signifikant mit dem Auftreten von Fernmetastasen, Lymphknotenrezidiven oder Lokalrezidiven verbunden sind.

**Ergebnisse:**

Die Tumorgröße in cm war signifikant höher bei Patienten mit Lokalrezidiv (Mittelwert 5,5), gefolgt von Fernmetastasen (Mittelwert 4,0), Lymphknotenrezidiv (Mittelwert 3,1) und rezidivfreie Patienten (Mittelwert 3,0), p<0,0001. Die Diffusionskapazität für Kohlenmonoxid (DLCO) in % war bei Patienten ohne Rezidiv signifikant höher (72,9) und nahm bei Patienten mit Fernmetastasierung (67,0), Lokalrezidiv (66,7) und Lymphknotenrezidiv (65,8) ab, p=0,01. Es gab keine signifikanten Unterschiede bei den postoperativen Komplikationen, dem chirurgischen Zugang oder der Anzahl an untersuchten Lymphknoten. Eine Tumorgröße ≥4,65 cm wurde als unabhängiger Faktor für ein Lokalrezidiv identifiziert.

**Schlussfolgerung:**

Bei Patienten mit NSCLC ≥4,65 cm, was dem UICC-Stadium IIA und höher entspricht, sollte eine multimodale Therapie diskutiert werden. Der chirurgische Zugang hat keinen Einfluss auf das Rezidiv.

## Introduction

The medical research and treatment of lung cancer is of significant importance due to the fact that lung cancer is the most common diagnosis in cancer-related deaths and has a poor overall survival rate 1 . Surgical therapy is the preferred treatment modality for lung cancer, with the objective of improving overall survival and ensuring that patients are tumor-free postoperatively 2 . However, even if this objective is met, 20% of patients who actually had an early tumor stage (negative resection margin, negative lymph nodes, absence of distant metastases) are nevertheless diagnosed with a tumor recurrence within two years 3 4 5 6 . Therefore, a primary objective is to achieve a substantial reduction in the recurrence rate of lung cancer and to identify predictors that influence its recurrence.

The risk factors published by some research groups as an indicator of recurrence were not all robust and were partly refuted by other research findings 4 7 8 9 10 11 . While certain groups have developed a clinicopathologic prediction model for postoperative recurrence for very early tumor stages 7 , others have been unable to develop a model based on five well-known clinical risk factors (tumor size and grade, visceral pleural and lymphovascular invasion and sublobar resection) 3 . To date there is a paucity of clear clinical factors predicting recurrence in postoperative early stages (N0, R0, M0), highlighting the complexity of this issue. Consequently, there is an absence of clear recommendations regarding suitable pre- or postoperative therapy in the absence of metastasis. According to the National Comprehensive Cancer Network, the boundary is determined between stage IIA and IIB. Patients with postoperative stage IIA (T2b, N0, M0) should either be observed or receive adjuvant therapy. Patients diagnosed with stage IIB (T3, N0, M0) and above should receive adjuvant therapy 12 .

The aim of the study was to analyze in a very large cohort whether there are certain risk factors for postoperative tumor recurrence in order to derive possible recommendations that reduce the risk of recurrence. In addition, the question is whether there are factors that lead to a recommendation for neoadjuvant or adjuvant therapy, even if the postoperative pathological findings show an early tumor stage (N0, R0, M0). The inclusion criteria encompassed all T-stages (T1–T4), while patients with nodal involvement at diagnosis or distant metastases were excluded from the study.

## Methods

### Study Design, Patient Cohort and Data Collection

In this retrospective analysis, we used data of all non-small cell lung cancer patients undergoing lobectomy through thoracotomy or VATS at the Lung Cancer Centre Munich between 2011 and 2020. Preoperatively, all patients were staged according to the current National Comprehensive Cancer Network (NCCN) guidelines and were discussed at the specific tumor board. Patients underwent pre- or intraoperative bronchoscopy, pathological lymph node evaluation and FDG-PET/CT scan. Meanwhile, the method of choice for lymph node evaluation was EBUS-TBNA. Historically, a mediastinoscopy was performed in cases where there was a suspicion of lymph node involvement. Mediastinoscopy is still the method of choice for patients with a clinically (FDG-PET/CT and/or CT) positive mediastinum and negative EBUS-TBNA for malignancy. Patients with clinical stage II and expected N0 status also receive a cranial MRI 12 .

Only patients who received a lobectomy were included in this study. Patients with re-lobectomy, nodal involvement at diagnosis (N1, N2 and N3), patients with distant metastases at diagnosis, positive R status and patients with missing pathological status were excluded from the study, as were patients with segmental resections or wedge resections. In patients who underwent more than one lobectomy due to a lung tumor within the study period (second carcinoma), only the initial resection was included in the analysis.

The patient population was divided into four groups based on their recurrence status. A systematic differentiation was employed, classifying cases into the following categories: distant recurrence, lymph node recurrence, local recurrence, and no recurrence. A comprehensive analysis was conducted, encompassing patient characteristics, tumor characteristics, perioperative outcomes, therapeutic interventions, and complications. A set of substantial discrepancies were identified. All information in the dataset was derived from electronic patient records and archived charts. This dataset encompassed patient characteristics, including age at resection, body mass index (BMI), comorbidities, gender, smoking status, performance status according to the American Society of Anesthesiologists risk classification (ASA), spirometry and blood test results. Additionally, the dataset included information regarding the operation, postoperative therapy and complications. The tumor characteristics encompassed a comprehensive array of parameters, including clinical and pathological tumor stage, histological type, tumor location, as well as tumor grading, lymphovascular space invasion (L-status) and vascular invasion (V-status).

### Perioperative outcomes, complications and multimodal therapy

In order to facilitate a meaningful comparison between the groups, a preoperative functional status analysis was conducted, with the parameters of vital capacity (VC), forced expiratory volume in 1 second (FEV _1_ ) and diffusion capacity of the lung for carbon monoxide (DLCO). Furthermore, blood tests at staging and after surgery were analyzed. The analysis focused on key parameters, including the concentration of hemoglobin (in g/L), the serum creatinine concentration (in mg/dL), the level of C-reactive protein (CRP) (in mg/dL), the total leucocyte count (in G/L), and the changes in hemoglobin levels before and after the surgical intervention. In order to draw comparisons between the groups with regard to the surgical therapy, we analyzed the surgical approach (VATS versus thoracotomy), the number of lymph nodes assessed during surgery, the duration of surgery in minutes, length of hospitalization after surgery in days (LOS), the blood loss in ml and the total volume of the resected lung in ml.

In the context of multimodal therapy, a distinction was made between neoadjuvant and adjuvant therapy, with each of these categories further subdivided into chemotherapy and radiotherapy modalities. The indications for neoadjuvant therapy were determined by the tumor size and its position relative to critical anatomical structures, with the objective of ensuring operability. The indications for adjuvant therapy were also determined by tumor size (with adjuvant chemotherapy or adjuvant radiotherapy of the thoracic wall in the case of intraoperative infiltration or for tumors that reached close to the resection margin). All patients had no N1 or R1 situation (only N0, R0 patients were included). The indications for adjuvant therapy were decided on a case-by-case basis by the interdisciplinary tumor board and were carried out as prophylactic adjuvant therapy.

Postoperative complications included the occurrence of pneumonia, the formation of a fistula lasting more than five days, the necessity for repeated postoperative thoracal puncture or repeated placement of a chest tube due to pleural effusion, postoperative bronchoscopy, cardiac arrhythmia and the requirement for blood transfusion.

### Categorization of variables and handling of missing data

The histological types were categorized into the following: adenocarcinoma (ACC), squamous-cell carcinoma (SCC), and neuroendocrine carcinoma (including carcinoids and large-cell neuroendocrine carcinomas) (NEC). The remaining histological types were grouped into the category designated “other histology”.

BMI was missing for a few patients, so we used multiple imputation to fill in the missing values. We created a category called “unknown” for the categorial outcome.

### Statistical Analysis

Patient characteristics are presented as mean values with standard deviation (SD) for metric variables and absolute and relative frequencies for categorical variables. A comparison was made between the numerical outcomes of patients with local, distant, lymph node and no recurrence using analysis of variance (ANOVA), and categorical outcomes using Chi2-test or Fisher’s exact test when cell numbers were <6. Logistic regression models were applied for the purpose of identifying factors that exhibited a significant association with both the occurrence of a recurrence and the occurrence of a local recurrence. For the multivariate logistic regression, the selection of variables was based on predefined inclusion criteria. Specifically, we included variables that are known risk factors according to the literature or are generally recognized as clinically relevant. Furthermore, variables that demonstrated a statistically significant association in the univariate analysis (p<0.05) were also given consideration. The multivariate model incorporated a total of 10 variables. It is acknowledged that this figure constitutes a relatively high number of variables in relation to the 107 events observed. Nonetheless, in accordance with the prevailing convention for logistic regression, which stipulates the incorporation of approximately one variable for every 10 events, the number of variables remains within an acceptable range 13 . The results of the logistic regression are presented as odds ratios (OR) with p-values. The threshold for significance was set at alpha <0.05. The determination of the optimal cut-off value for tumor size in cm and local recurrence, as well as DLCO in percentage and any recurrence, were determined using receiver operating characteristic curves (ROC) with area under the curve (AUC) in conjunction with specificity and sensitivity.

The data analysis was conducted utilizing the R Version 4.0.0 and RStudio Version 1.4 software. The tables and the figures were created in RStudio and Microsoft Excel.

## Results

### Patient population and tumor characteristics

A total of 1680 lobectomies were carried out at our center between the years 2011 and 2020. Following the exclusion of patients who had undergone re-lobectomies (n=25), those with nodal involvement or missing information on nodal involvement (n=831), patients with positive M status (n=32), patients with positive R status (n=7), patients with missing information on comorbidities (n=2), and patients with missing information on recurrence status (n=2), the analysis was conducted on data from 781 patients. Of those 781 patients, 674 demonstrated no recurrence, 45 patients exhibited distant metastases, 14 patients were affected by lymph node involvement and 48 patients experienced local recurrences in the subsequent course of the disease.

**Fig. 1:**
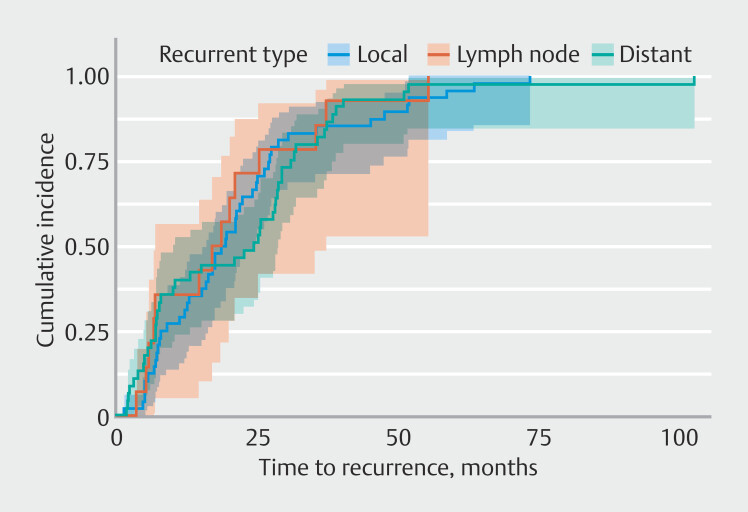
Cumulative incidence of tumor recurrence following surgical resection of lung cancer. The curves display the cumulative incidence function for patients with local, lymph node or distant recurrence. The y-axis represents the cumulative probability of recurrence, and the x-axis denotes time after surgery (months). Shaded areas indicate the 95% confidence intervals of the respective cumulative incidence estimates.

The median time to recurrence for all tumor stages included was 19.5 months. When the data were disaggregated into subgroups, recurrence was observed in the group with distant metastases after a median of 24.37 months, in the lymph node recurrence group after 17.72 months, and in the group with local recurrence after 18.95 months. There is no significant difference in the respective subgroups until recurrence occurs (p=0.785). Fig. 1 presents the cumulative incidence of tumor recurrence, divided into the three subgroups mentioned above.

The distribution of gender differed significantly across the four recurrence groups (p<0.01). The comorbidity of fibrosis was found to be significantly higher in patients with a local recurrence (p=0.04). No significant differences were identified in age, BMI, Charlson Comorbidity Index (CCI), smoking status, American Society of Anesthesiologists risk classification (ASA), or comorbidities, apart from the fibrosis already reported.

In relation to the characteristics of the tumors, a statistically significant correlation was identified between tumor size, measured in cm, and the presence of local recurrence. Patients exhibiting a local recurrence demonstrated a mean tumor size of 5.5 cm, followed by distant recurrence (mean 4.0 cm), lymph node recurrence (mean 3.1 cm) and finally patients with no recurrence (mean 3.0 cm), p<0.0001. A similar sequence was observed for the T4 status, which was significantly more prevailed in patients with local recurrence (18.8%) and decreased with distant recurrence (8.9%), no recurrence (2.1%) and lymph node recurrence (0.0%) (p<0.0001). Conversely, T1 status exhibited a significantly lower frequency of local recurrence (18.8%) and an increased frequency of lymph node recurrence (28.6%), distant recurrence (33.3%) and no recurrence (51.6%). A total of 376 patients were diagnosed with T1 tumors (49.3%), 287 with T2 (37.6%), 73 with T3 (9.6%) and 27 with T4 tumors (3.5%).

With the exception of NEC (p=0.001), which occurred significantly more frequently in patients without recurrence, no differences were observed between the respective recurrence groups with regard to histological type. No significant variations were observed in the remaining tumor characteristics. Table 1 and Table 2 present a summary of all patients and tumor characteristics.

**Table:** **Table 1** Patient characteristics of study population. Patient characteristics of lung cancer patients with lobectomy and recurrence. Means with standard deviation of numerical variables and absolute and relative frequency of categorical variables.

	distant recurrence (n=45)		lymph node recurrence ( n=14)		local recurrence (n=48)		no recurrence (n=674)		p-value	
	median	95%CI	median	95%CI	median	95%CI				
temporal interval until recurrence occurs (month)	24.37	[10.0, 28.7]	17.72	[6.7, 37.2]	18.95	[15.8, 24.1]			0.785	
	mean	sd	mean	sd	mean	sd	mean	sd		
age in years	66.7	7.6	68.2	6.6	66.6	10.8	66.5	11.0	0.93	
Body Mass Index	25.1	4.7	25.5	3.7	25.3	4.7	26.1	4.8	0.53	
Charlson Comorbidity Index	3.4	1.7	3.7	1.4	3.6	2.0	3.6	2.1	0.82	
	n	%	n	%	n	%	n	%	p-value	
**sex**										
male	22	48.9%	12	85.7%	31	64.6%	328	48.7%		
female	23	51.1%	2	14.3%	17	35.4%	346	51.3%	0.01	*
**current smoker**										
yes	14	31.1%	5	35.7%	13	27.1%	174	25.8%		
no	31	68.9%	9	64.3%	34	70.8%	488	72.4%		
unknown	0	0.0%	0	0.0%	1	2.1%	12	1.8%	0.78	
**ASA**										
1	3	6.7%	0	0.0%	1	2.1%	27	4.0%		
2	8	17.8%	2	14.3%	12	25.0%	186	27.6%		
3	29	64.4%	10	71.4%	30	62.5%	442	65.6%		
unknown	5	11.1%	2	14.3%	5	10.4%	19	2.8%	0.02	*
myocardial infarction	1	2.2%	2	14.3%	2	4.2%	35	5.2%	0.37	
peripheral arterial disease	4	8.9%	1	7.1%	2	4.2%	44	6.5%	0.74	
cerebrovascular disease	2	4.4%	1	7.1%	1	2.1%	48	7.1%	0.56	
chronic obstructive pulmonary disease	10	22.2%	7	50.0%	19	39.6%	193	28.6%	0.08	
asthma bronchiale	3	6.7%	1	7.1%	1	2.1%	51	7.6%	0.56	
collagenosis	0	0.0%	0	0.0%	0	0.0%	3	0.4%	1.00	
gastroduodenal ulcer	1	2.2%	0	0.0%	3	6.3%	16	2.4%	0.36	
mild liver disease	0	0.0%	0	0.0%	1	2.1%	16	2.4%	0.89	
diabetes without end-organ damage	5	11.1%	1	7.1%	5	10.4%	90	13.4%	0.91	
diabetes with end-organ damage	2	4.4%	0	0.0%	1	2.1%	14	2.1%	0.51	
moderate to severe liver disease	0	0.0%	0	0.0%	0	0.0%	7	1.0%	1.00	
moderate to severe kidney insufficiency	0	0.0%	0	0.0%	3	6.3%	31	4.6%	0.43	
metastatic tumor	1	2.2%	0	0.0%	1	2.1%	8	1.2%	0.46	
leukaemia	0	0.0%	0	0.0%	0	0.0%	3	0.4%	1.00	
lymphoma	0	0.0%	0	0.0%	1	2.1%	8	1.2%	0.74	
coronary heart disease	5	11.1%	3	21.4%	11	22.9%	106	15.7%	0.38	
atrial fibrillation	4	8.9%	2	14.3%	7	14.6%	54	8.0%	0.25	
arterial hypertension	25	55.6%	9	64.3%	26	54.2%	387	57.4%	0.91	
pulmonary hypertension	0	0.0%	0	0.0%	1	2.1%	9	1.3%	0.77	
fibrosis	1	2.2%	0	0.0%	2	4.2%	3	0.4%	0.04	*
prior thoracic surgery	4	8.9%	1	7.1%	5	10.4%	71	10.5%	1.00	
CI=confidence interval, sd=standard deviation, ASA=American Society of Anesthesiologists risk classification, *=Variable with statistical significance										

**Table:** **Table 2** Tumor characteristics of study population. Tumor characteristics of lung cancer patients with lobectomy and recurrence. Means with standard deviation of numerical variables and absolute and relative frequency of categorical variables.

	distant recurrence (n=45)		lymph node recurrence ( n=14)		local recurrence (n=48)		no recurrence (n=674)		p-value	
	mean	sd	mean	sd	mean	sd	mean	sd		
tumor size in cm	4.0	2.7	3.1	1.7	5.5	3.7	3.0	2.0	<0.0001	*
	n	%	n	%	n	%	n	%	p-value	
**t status**										
T1	15	33.3%	4	28.6%	9	18.8%	348	51.6%	<0.0001	*
T2	16	35.6%	7	50.0%	22	45.8%	242	35.9%	0.44	
T3	5	11.1%	3	21.4%	8	16.7%	57	8.5%	0.07	
T4	4	8.9%	0	0.0%	9	18.8%	14	2.1%	<0.0001	*
**histological type**										
adenocarcinoma	26	57.8%	8	57.1%	35	72.9%	390	57.9%	0.24	
SCC	15	33.3%	4	28.6%	11	22.9%	159	23.6%	0.48	
NEC	0	0.0%	2	14.3%	2	4.2%	104	15.4%	0.001	*
*LCNEC*	*0*	*0.0%*	*2*	*100%*	*1*	*50.0%*	*24*	*23.8%*		
*Typical carcinoid*	*0*	*0.0%*	*0*	*0.0%*	*0*	*0.0%*	*70*	*69.3%*		
*Atypical carcinoid*	*0*	*0.0%*	*0*	*0.0%*	*1*	*50.0%*	*10*	*9.9%*		
other	4	8.9%	0	0.0%	0	0.0%	21	3.1%	0.13	
**G**										
1	1	2.2%	0	0.0%	4	8.3%	59	8.8%	0.39	
2	24	53.3%	4	28.6%	27	56.3%	352	52.2%	0.33	
3	17	37.8%	7	50.0%	15	31.3%	189	28.0%	0.16	
4	0	0.0%	0	0.0%	1	2.1%	6	0.9%	0.65	
unknown	3	6.7%	3	21.4%	1	2.1%	68	10.1%		
**L**										
0	36	80.0%	12	85.7%	33	68.8%	519	77.0%		
1	9	20.0%	2	14.3%	13	27.1%	125	18.5%	0.62	
unknown		0.0%	0	0.0%	2	4.2%	30	4.5%		
**V**										
0	39	86.7%	11	78.6%	36	75.0%	560	83.1%		
1	6	13.3%	3	21.4%	10	20.8%	84	12.5%	0.40	
unknown	0	0.0%	0	0.0%	2	4.2%	30	4.5%		
sd=standard deviation, T=tumor, SCC=squamous-cell carcinoma, NEC=neuroendocrine carcinoma, LCNEC=large cell neuroendocrine carcinoma of the lung, G=grading, L=lymphovascular space involvement, V=vascular invasion, *=Variable with statistical significance										

### Perioperative outcomes, multimodal therapy and complications

The findings of this study demonstrated that the DLCO percentage was considerably elevated in patients who did not experience recurrence (72.9%), while it exhibited a decline in patients with distant recurrence (67.0%), local recurrence (66.7%), and lymph node recurrence (65.8%), with a p-value of 0.01. A higher preoperative hemoglobin level was observed in patients who experienced lymph node recurrence (p=0.04). Furthermore, a significant increase in leucocyte levels was noted in patients with distant recurrence (p=0.03). Statistically significant variations were not identified in the operation data. For instance, there were no statistically significant differences observed in VATS or thoracotomy, the number of assessed lymph nodes, the duration of surgery, or the quantity of blood loss (in ml). The mean number of lymph nodes assessed was 17.2 in patients with distant recurrence, 15.9 in patients with lymph node recurrence, 15.0 in patients with no recurrence and 14.2 in cases of local recurrence.

A total of 45 patients received multimodal therapy, comprising 16 patients who received neoadjuvant therapy and 29 patients who received adjuvant therapy.

Patients who subsequently developed distant recurrence exhibited a significantly higher probability of receiving neoadjuvant therapy (p=0.01). This outcome was mostly driven by a greater proportion of neoadjuvant chemotherapy (p=0.004). A statistically significant association was identified between the application of adjuvant therapy and patients who were faced with the occurrence of lymph node recurrence at a later stage of the disease (p=0.04). This outcome was primarily attributable to a higher proportion of adjuvant radiotherapy (p=0.03).

The analysis conducted did not reveal any significant differences in postoperative complications. The perioperative outcomes, therapy and complications are outlined in Table 3 .

**Table:** **Table 3** Perioperative outcomes, therapy and complications. Perioperative outcomes, therapy and complications of lung cancer patients with lobectomy and recurrence. Means with standard deviation of numerical variables and absolute and relative frequency of categorial variables.

	distant recurrence (n=45)		lymph node recurrence ( n=14)		local recurrence (n=48)		no recurrence (n=674)			
	mean	sd	mean	sd	mean	sd	mean	sd	p-value	
**spirometry**										
VC in %	93.0	17.8	89.9	15.4	90.7	19.6	94.6	17.2	0.25	
FEV1%	85.3	20.0	74.5	17.4	79.7	22.8	84.5	20.0	0.07	
DLCO%	67.0	18.2	65.8	23.0	66.7	20.1	72.9	18.0	0.01	*
**blood at staging**										
hemoglobin	13.5	1.4	14.6	1.0	13.6	2.0	13.6	1.7	0.04	*
creatinine	1.2	1.0	1.0	0.2	1.1	0.3	1.0	0.6	0.05	
c-reactive protein	2.5	7.1	1.2	2.4	1.1	2.2	1.0	2.3	0.41	
leucocytes	9.1	3.6	8.2	1.6	7.5	2.4	7.9	4.2	0.03	*
**blood post OP**										
hemoglobin	12.0	1.6	13.0	1.4	12.0	1.9	12.0	1.6	0.15	
creatinine	1.0	0.7	0.9	0.2	1.1	1.3	0.9	0.9	0.25	
c-reactive protein	8.9	6.7	7.1	6.8	6.9	5.1	7.9	6.4	0.43	
leucocytes	13.7	5.2	13.4	3.5	12.3	5.1	12.2	4.3	0.13	
change in hemoglobin pre and post OP	1.5	1.14	1.67	0.825	1.62	0.958	1.59	1.27	0.82	
operation	n	%	n	%	n	%	n	%	p-value	
VATS	19	42.2%	6	42.9%	18	37.5%	297	44.1%		
thoracotomy	26	57.8%	8	57.1%	30	62.5%	377	55.9%	0.84	
# of assessed lymph nodes	17.2	9.16	15.9	7.97	14.2	10.8	15	7.68	0.20	
length of surgery in min	178.3	53.0	154.5	55.3	175.4	52.6	181.6	53.8	0.22	
LOS	13.6	5.2	12.1	2.3	14.4	5.6	14.0	7.4	0.55	
blood loss in ml	476.2	446.3	500.0	419.5	383.3	170.5	433.1	513.0	0.78	
total volume of specimen in ml	1637.5	1242.8	1677.3	1210.2	1471.4	876.2	1270.7	778.8	0.08	
multimodal therapy	n	%	n	%	n	%	n	%	p-value	
neoadjuvant chemotherapy	5	11.1%	0	0.0%	0	0.0%	9	1.3%	0.004	*
neoadjuvant radiotherapy	1	2.2%	0	0.0%	0	0.0%	5	0.7%	0.41	
neoadjuvant therapy	5	11.1%	0	0.0%	0	0.0%	11	1.6%	0.01	*
adjuvant chemotherapy	3	6.7%	0	0.0%	3	6.3%	15	2.2%	0.10	
adjuvant radiotherapy	0	0.0%	2	14.3%	0	0.0%	7	1.0%	0.03	*
adjuvant therapy	3	6.7%	2	14.3%	3	6.3%	21	3.1%	0.04	*
complications	n	%	n	%	n	%	n	%	p-value	
pneumonia	6	13.3%	1	7.1%	8	16.7%	114	16.9%	0.84	
fistula >5 days after surgery	3	6.7%	0	0.0%	6	12.5%	59	8.8%	0.59	
repeated thoracic puncture/	2	4.4%	0	0.0%	4	8.3%	54	8.0%	0.80	
**chest tube placement**										
postoperative bronchoscopy	7	15.6%	0	0.0%	2	4.2%	81	12.0%	0.17	
cardiac arrhythmia	3	6.7%	2	14.3%	3	6.3%	63	9.3%	0.71	
blood transfusion	4	8.9%	0	0.0%	2	4.2%	19	2.8%	0.06	
sd=standard deviation, VC=vital capacity, FEV1=Forced Expiratory Volume in 1 second, DLCO=diffusing capacity of the lung for carbon monoxide, VATS=video-assisted thoracoscopic surgery, LOS=length of hospital stay after surgery in days, *=Variable with statistical significance										

### Multivariate regression analysis

Multivariate logistic regression analysis demonstrated a significantly elevated risk of recurrence for T2 status in comparison to T1 status (OR=2.18, p=0.005) and for T4 status in comparison to T1 status (OR=15.64, p=0.0001). In relation to the histological classification of the tumor, patients diagnosed with NEC exhibited a reduced probability for recurrence in comparison to those diagnosed with ACC (OR 0.14, p=0.01). Other histological types were not significantly different. An increase in DLCO percentage was found to be significantly associated with a reduced risk of recurrence (odds ratio [OR]=0.98, p=0.03). In terms of local recurrence in comparison to no local recurrence, a significantly higher risk for local recurrence was identified in T2, T3 and T4 status in comparison to T1 status (HR=4.2 vs. 4.3 vs. 53.83, p=0.002 vs. p=0.02 vs. p<0.0001). All results of the study are presented in Table 4 **A,B** .

**Table:** **Table 4** Results from Logistic Regression analysis.

	OR	coef	se	z-value	p-value	
**A Recurrence vs. no recurrence**						
female vs. male	0.77	–0.27	0.26	–1.02	0.31	
ASA 2 vs. ASA 1	0.39	–0.95	0.67	–1.42	0.15	
ASA 3 vs. ASA 1	0.44	–0.82	0.64	–1.28	0.20	
ASA missing vs. ASA 1	0.68	–0.38	0.85	–0.45	0.65	
fibrosis vs. no fibrosis	2.85	1.05	0.90	1.17	0.24	
T2 vs. T1	2.18	0.78	0.28	2.82	0.005	*
T3 vs. T1	2.24	0.81	0.41	1.96	0.05	
T4 vs. T1	15.64	2.75	0.51	5.42	<0.0001	*
NEC vs. adenocarcinoma	0.14	–1.96	0.75	–2.60	0.01	*
other histology vs. adenocarcinoma	0.46	–0.77	0.80	–0.96	0.34	
SCC vs. adenocarcinoma	0.92	–0.08	0.28	–0.28	0.78	
adjuvant therapy	1.96	0.67	0.51	1.33	0.18	
DLCO%	0.98	–0.02	0.01	–2.19	0.03	*
hemoglobin at staging	1.13	0.12	0.07	1.66	0.10	
creatinine at staging	1.33	0.29	0.16	1.84	0.07	
leukocytes at staging	0.99	–0.01	0.02	–0.47	0.64	
**B Local recurrence vs. no local recurrence**						
female vs. male	0.66	–0.41	0.38	–1.07	0.28	
ASA 2 vs. ASA 1	0.49	–0.71	1.13	–0.63	0.53	
ASA 3 vs. ASA 1	0.43	–0.84	1.12	–0.75	0.45	
ASA missing vs. ASA 1	0.41	–0.88	1.50	–0.59	0.56	
fibrosis vs. no fibrosis	2.56	0.94	1.09	0.86	0.39	
T2 vs. T1	4.20	1.44	0.46	3.10	0.002	*
T3 vs. T1	4.30	1.46	0.64	2.29	0.02	*
T4 vs. T1	53.83	3.99	0.68	5.86	<0.0001	*
NEC vs. adenocarcinoma	0.13	–2.03	1.08	–1.88	0.06	
other histology vs. adenocarcinoma	0.00	–15.44	843.22	–0.02	0.99	
SCC vs. adenocarcinoma	0.71	–0.34	0.43	–0.80	0.43	
adjuvant therapy	1.53	0.42	0.75	0.56	0.57	
DLCO%	0.98	–0.02	0.01	–1.83	0.07	
hemoglobin at staging	1.10	0.10	0.10	0.99	0.32	
creatinine at staging	1.39	0.33	0.21	1.55	0.12	
leukocytes at staging	0.88	–0.12	0.08	–1.52	0.13	
Results from Logistic regression analysis of local recurrence vs. no recurrence (A), local recurrence vs. no local recurrence (B), OR=Odds ratio, coef=coefficient of variation, se=standard error, ASA=American Society of Anesthesiologist risk classification, T=tumor, NEC=neuroendocrine carcinoma, SSC=squamous-cell carcinoma, DLCO=diffusion capacity of the lung for carbon monoxide, *=Variable with statistical significance						

### Cut-off values for DLCO percentage and tumor size in cm

**Fig. 2:**
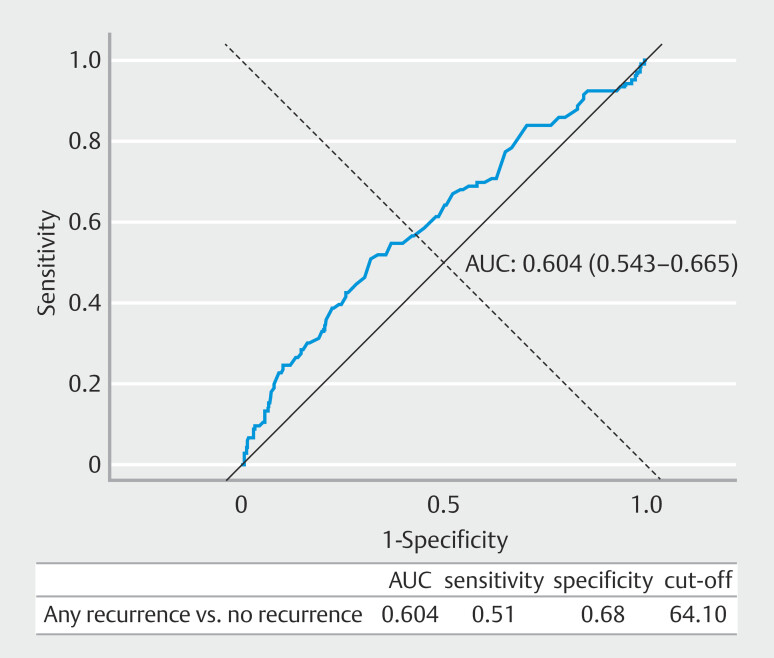
ROC curve DLCO%; With this ROC curve we determined an optimal cut-off point of 64.10% for identifying patients with a higher risk for a recurrence. The AUC was 0.604, sensitivity of 0.51 and a specificity 0.68. AUC=Area under the curve, ROC=receiver operating characteristic curves.

The determination of an optimal cut-off point for identifying patients with a higher risk of recurrence was enabled by the DLCO percentage ROC curve. This analysis revealed that a cut-off point of 64.1% or lower would be effective in this regard. The Area Under the Curve (AUC) was determined to be 0.604, with a sensitivity of 0.51 and a specificity of 0.68. The ROC curve is illustrated in Fig. 2 .

**Fig. 3:**
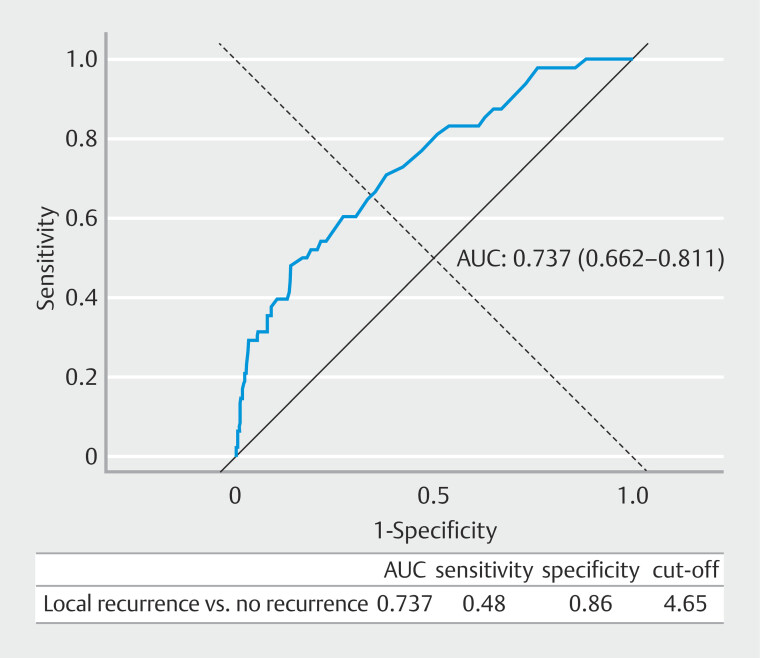
ROC curve tumor size; With this ROC curve we determined an optimal cut-off point of 4.65 cm for identifying patients with a higher risk for a local recurrence. The AUC was 0.737, sensitivity of 0.48 and a specificity 0.86. AUC=Area under the curve, ROC=receiver operating characteristic curves.

In terms of the tumor size, the implementation of the ROC curve enabled the determination of an optimal cut-off point of 4.65 cm for the identification of patients exhibiting a higher risk of local recurrence. The area under the curve (AUC) was determined to be 0.737, with a sensitivity of 0.48 and a specificity of 0.86. The ROC curve is illustrated in Fig. 3 .

## Discussion

This study is large-scale, with a final population of 781 postoperative patients up to stage IIIA. The studyʼs findings substantiated the existence of independent risk factors associated with the potential for recurrence. Patients who had experienced a recurrence demonstrated increased tumor size and reduced DLCO levels in comparison to those who did not experience recurrence. The investigation revealed no significant correlation between the recurrence rate and either the surgical approach (VATS or thoracotomy) or the number of lymph nodes assessed. A plethora of discussions have emerged concerning minimally invasive surgery and thoracotomy. In a meta-analysis on the subject of recurrence, Zhang et al. reported that both systemic and locoregional recurrence rates were found to be significantly lower in cases involving VATS 14 . Yun et al. were unable to demonstrate a significant difference between VATS and thoracotomy for tumor sizes >5cm 15 . The conclusion drawn from the examination of our patient population (up to stage IIIA) was that there was no difference in the impact of VATS and open surgery on recurrence rate. With regard to the number of lymph nodes assessed, no correlation was found between this and the occurrence of recurrences. However, it should be noted that our center has a higher rate of lymph node removal during surgery compared to other institutions 16 17 18 . As recently published, we perform a standardized lymphadenectomy in accordance with the recommendations whenever possible 19 20 . The removal of a large number of lymph nodes can prevent potential lymph node metastasis and facilitate more accurate staging. The substantial number of lymph nodes may provide a rationale for the low incidence of lymph node recurrences (n=14) observed in the present study. However, further studies comparing patients with a low number of examined lymph nodes with those with a higher number of examined lymph nodes are required to prove this.

The present study identified two independent risk factors for postoperative recurrence: reduced DLCO and tumor size. However, subsequent analyses showed that reduced DLCO has only limited clinical significance for predicting recurrence. This hypothesis was supported by the odds ratio of 0.98. To further differentiate the significance of DLCO, a ROC curve was used in conjunction with specificity and sensitivity. The optimal cut-off value for DLCO was determined to be 64.10%, with an AUC value of 0.604, sensitivity of 0.51, and specificity of 0.68. This demonstrated that, while DLCO could be identified as a significant factor, its clinical significance would be minimal in practice. In a cohort of patients with a DLCO of 64.10% or less, 51% of the patients experienced a recurrence. Conversely, 68% of patients with a DLCO greater than 64.10% were correctly classified as non-recurrent. Despite its lack of reliability and clinical relevance, DLCO is statistically a predictor of recurrence. For this reason, we considered it appropriate to discuss possible causes of the increased recurrence rate with reduced DLCO. Firstly, the reduced DLCO could be indicative of a chronic lung disease, as this is often associated with a reduced DLCO. It is known that such diseases are associated with an elevated risk of lung cancer 21 22 . In that regard Tzouvelekis et al. described that 10% incidence of lung cancer in patients diagnosed with idiopathic pulmonary fibrosis 23 . Nagai et al. reported on the same subject a value of 31.3% 14 . It is hypothesized that the disease may manifest in the scarred fibrotic regions, which are colloquially referred to as scar carcinoma 22 24 . The investigation into whether chronic lung diseases such as idiopathic pulmonary fibrosis or chronic obstructive pulmonary disease are also a risk factor for the development of recurrence is yet to be completed. For instance, do these phenomena manifest postoperatively in residual fibrotic regions or in scarring that develops in the areas of resection? It is important to note that such a potential scenario would only apply to local recurrences. This assertion is supported by the findings of the present study, which have proven that the number of patients with fibrosis is significantly higher in the local recurrence group (p=0.04). Another potential explanation for the impact of DLCO is the correlation of DLCO with the histopathological aggressiveness of lung cancer. Ozeki et al. described a significant correlation between low DLCO in adenocarcinoma patients and carcinogenesis and progression 25 .

Furthermore, the presence of poorly differentiated tumors, high scar grade and nuclear atypia can also serve as indicators of an elevated risk of recurrence. Moreover, the tumor size, which was identified as the second risk factor, could provide a rationale for the diminished DLCO observed in patients who have experienced recurrence. The present study demonstrated a negative correlation between DLCO and tumor mass, suggesting that patients exhibiting reduced DLCO levels may be predisposed to elevated risk of recurrence, attributable to the presence of larger tumors, which lead to reduced DLCO.

It can be concluded that a potential correlation between reduced DLCO and tissue-sparing resection, respectively a recurrence due to a reduced resection distance, can be disregarded, as the present study is solely based on patients who underwent lobectomy. In certain cases, a tissue-sparing procedure may be employed in lieu of a lobectomy, particularly when patients are deemed to lack the capacity to withstand the postoperative recovery process due to pre-existing pulmonary dysfunction.

In summary, further analysis is needed to gain a more detailed understanding of the pathomechanism that explains the association between reduced DLCO and postoperative recurrence.

The second independent risk factor identified, tumor size, has previously been described as a predominant risk factor in a number of other studies 26 27 28 . In the study by Takahashi et al. (2014), the occurrence of recurrences was found to have a sensitivity of 34% and a specificity of 68.2% for pathological stage II. The area under the curve (AUC) was not specified in this study 28 . As published by Isaka et al., the presence of a tumor measuring >2.4 cm has been identified as a potential indicator for the likelihood of subsequent recurrence. However, it should be noted that a ROC analysis has not been included in this study 27 . In our present analysis, the tumor size was found to be a significant factor for all recurrence groups. Given the local recurrence group (48 patients) exhibited the largest tumors and, equivalently, the most patients with T4 stages, a closer look at the tumor size in this group was warranted. In the logistic regression, T2, T3 and T4 stages demonstrated a significantly elevated risk of local recurrence in comparison to the T1 stage (OR 4.2–53.83). Subsequently, a ROC curve was created for the purpose of determining a cut-off value. The optimal cut-off point for identifying patients at increased risk of local recurrence was determined to be 4.65cm, with a sensitivity of 48% and a specificity of 86%. The AUC of 0.74 emphasizes the statistical robustness of the analysis.

As in the study group led by Takahashi et al., the cut-off point is UICC stage II. According to the 9 ^th^ edition of the Tumor Node Metastasis (TNM) staging system and The Union for International Cancer Control (UICC), patients with a tumor size of 4–5 cm and no lymph node involvement are classified as UICC stage IIA 29 .

In the event that patients with a tumor size of ≥4.65 cm had been treated with a multimodal therapy, 48% of subsequent local recurrences would have already been treated. Furthermore, only 14% would have been treated in cases where no local recurrence had occurred later. Assuming that, as described in the literature, 20% of patients with postoperative node-negative, margin-negative and distant metastasis-negative lung cancer develop recurrence within the first two years 4 5 , or 33% within five years 8 , the high number of recurrences could be reduced by directly offering multimodal therapy to those whose tumor is ≥4.65 cm. The National Comprehensive Cancer Network stipulates that patients with postoperative stage IIA who have a negative margin should either be observed or receive adjuvant therapy. It is recommended that, from stage IIB onwards, all patients receive adjuvant therapy 12 . The results of our study indicate that multimodal therapy should be discussed with every patient diagnosed with NSCLC ≥4.65 cm.

It is imperative to acknowledge the limitations of this study when interpreting the findings. Firstly, the present study is of a retrospective nature and was conducted in a single center. Secondly, the present study did not analyze overall survival (OS). In view of the considerable number of patients with whom we are engaged, it is not feasible to provide consistent follow-up and post-treatment after recurrence. Patients who developed a postoperative recurrence, in particular a lymph node recurrence or distant metastases, were subsequently treated not only at our institution but also by oncologists in private practices or received external stereotactic radiotherapy. Consequently, despite the recurrence being documented in our clinic and the procedure being discussed in the tumor board, there was a paucity of information regarding survival. Consequently, the decision was taken not to analyze the OS of all patients and the median survival after recurrence, as the results of such an analysis would not correspond to the actual results. Finally, we were unable to conduct any analyses of molecular markers. In the early years of the study period, tests for molecular markers were not yet available. This meant that many patients could not be tested at the time of their treatment. Unfortunately, it was not possible to perform these tests retrospectively. To avoid distorting the statistical results with missing data, we have decided to exclude the molecular markers.

## Conclusion

The findings of this study indicate that tumor size acts as an independent risk factor with clinical relevance for postoperative recurrence. It is recommended to consider multimodal therapy for patients diagnosed with NSCLC with a diameter of ≥4.65 cm, which corresponds to UICC stage IIA and above.

Despite its statistical significance, further analyses showed that DLCO is not particularly important in everyday clinical practice for predicting postoperative recurrence.

Furthermore, it has been proven that the surgical approach has no influence on the occurrence of recurrence.
